# Spatiotemporal receptive field structures in retinogeniculate connections of cat

**DOI:** 10.3389/fnsys.2013.00103

**Published:** 2013-12-09

**Authors:** Naofumi Suematsu, Tomoyuki Naito, Tomomitsu Miyoshi, Hajime Sawai, Hiromichi Sato

**Affiliations:** ^1^Laboratory of Cognitive and Behavioral Neuroscience, Department of Health and Sportsscience, Graduate School of Frontier Biosciences, Osaka UniversityOsaka, Japan; ^2^Laboratory of Cognitive and Behavioral Neuroscience, Department of Health and Sportsscience, Graduate School of Medicine, Osaka UniversityOsaka, Japan; ^3^Department of Integrative Physiology, Graduate School of Medicine, Osaka UniversityOsaka, Japan

**Keywords:** receptive field, retinal ganglion cell, lateral geniculate nucleus neuron, cross-correlation, cat

## Abstract

The spatial structure of the receptive field (RF) of cat lateral geniculate nucleus (LGN) neurons is significantly elliptical, which may provide a basis for the orientation tuning of LGN neurons, especially at high spatial frequency stimuli. However, the input mechanisms generating this elliptical RF structure are poorly defined. We therefore compared the spatiotemporal RF structures of pairs of retinal ganglion cells (RGCs) and LGN neurons that form monosynaptic connections based on the cross-correlation analysis of their firing activities. We found that the spatial RF structure of both RGCs and LGN neurons were comparably elliptical and oriented in a direction toward the *area centralis*. Additionally, the spatial RF structures of pairs with the same response sign were often overlapped and similarly oriented. We also found there was a small population of pairs with RF structures that had the opposite response sign and were spatially displaced and independently oriented. Finally, the temporal RF structure of an RGC was tightly correlated with that of its target LGN neuron, though the response duration of the LGN neuron was significantly longer. Our results suggest that the elliptical RF structure of an LGN neuron is mainly inherited from the primary projecting RGC and is affected by convergent inputs from multiple RGCs. We discuss how the convergent inputs may enhance the stimulus feature sensitivity of LGN neurons.

## Introduction

In the early visual system of mammals, visual information is received by the retina and then relayed to the primary visual cortex (V1) via the lateral geniculate nucleus (LGN) (Hubel and Wiesel, 1962, 1977). Through these stages, receptive field (RF) properties, such as orientation, spatial frequency (SF), and temporal frequency (TF) tuning, are successively elaborated, which expands the sensitivity for various visual features in neurons of the early visual system (Hubel and Wiesel, 1959, 1962; Enroth-Cugell and Robson, 1966; Campbell et al., 1969; Movshon et al., 1978; Derrington and Fuchs, 1979; Frishman et al., 1987).

In the LGN, it had been commonly believed that neurons exhibit only weak or no orientation selectivity, and their RFs are almost circular (Hubel and Wiesel, 1959, 1962). However, more recent studies have reported that LGN neurons exhibit moderate orientation sensitivity in cat (Soodak et al., 1987; Shou and Leventhal, 1989; Smith et al., 1990; Suematsu et al., 2012; Naito et al., 2013), mouse (Niell, 2013; Scholl et al., 2013; Zhao et al., 2013), and marmoset (Cheong et al., 2013) due to an elliptical RF structure (Soodak et al., 1987; Ahmed and Hammond, 1991; Suematsu et al., 2012).

There are at least two possible explanations for how the elliptically elongated RF structure of LGN neurons is generated. One is that the spatial RF structure of a retinal ganglion cell (RGC), which is an input source for LGN neurons, is also elongated such that the target LGN neuron directly reflects this structure. Rodieck and Stone (1965) and Hammond (1974), for example, reported the ellipticity of the spatial RF structure of cat RGCs. In addition, it is commonly thought that the connection between an RGC and LGN neuron is essentially one-to-one, because both the projecting RGC afferent and target LGN neuron have very similar spatial RF structures and properties (Soodak et al., 1987; Smith et al., 1990). The other explanation is that a single LGN neuron receives convergent inputs from multiple RGCs sharing in-line RF positions that elongate the RF of the LGN neuron (Tavazoie and Reid, 2000). Several studies have shown the possibility of convergent inputs in retinogeniculate connections (Usrey et al., 1999; Moore et al., 2011), which are important for creating a diversity of RF structures in different LGN neurons (Alonso et al., 2006). However, it is still unclear how convergent projections in retinogeniculate connections contribute to the spatial and temporal RF structure of LGN neurons.

To clarify the underlying mechanism involved, we simultaneously recorded the single-unit activities of RGCs and LGN neurons of cat during the presentation of two-dimensional dynamic dense noise stimuli and analyzed their RF structures, which were reconstructed by the reverse correlation technique using electrophysiologically-identified retinogeniculate connections. We found that RGCs and LGN neurons exhibited elliptical spatial RF structures, and that an RGC projection of the same response sign was the primary contributor to the generation of the RF center of the LGN neuron, while an RGC projection of the opposite response sign was responsible for enhancing the antagonistic surround. In addition, the temporal RF structure of an RGC was tightly correlated with its target LGN neuron, although the response duration was significantly shorter. These results suggest that the elongated RF of LGN neurons is mainly inherited from that of the primary-projecting RGC and that convergent inputs from multiple RGCs improve the stimulus feature sensitivity of LGN neurons, presumably by contributing to more efficient processing in the visual cortex.

## Methods

All experimental protocols were approved by the Research Ethics Committee of Osaka University. All animal procedures were performed in accordance with the National Institute of Health Guidelines for the Care and Use of Laboratory Animals and the Guidelines of the Animal Care Committee of the Osaka University Medical School. All efforts were made to reduce the number of animals used.

### Preparation

Four adult cats weighing 3.1–4.1 kg were used. Initially, atropine (0.1 mg, i.m.) was injected as premedication. Animals were anesthetized with ketamine hydrochloride (Ketalar; Sankyo, Tokyo, Japan; 25 mg/kg), placed in a stereotaxic head-holder, and then anesthetized with a mixture of N_2_O/O_2_ (1:1) after tracheal intubation. A catheter was placed in the femoral vein. During the entire experimental period (−48 h), to paralyze and maintain animals under artificial ventilation and to minimize eye movements, a mixture of N_2_O/O_2_ (1:1) was continuously supplied, and a solution of sodium pentobarbital (Somnopentyl; Kyoritsu, Tokyo, Japan; 1 mg/kg/h, i.v.) in Ringer's solution for anesthesia and a mixed solution of pancuronium bromide (Mioblock; MSD, Tokyo, Japan; 0.1 mg/kg/h, i.v.) and glucose in Ringer's solution for paralysis were continuously infused through the femoral vein at 0.5 and 1.5 ml/kg/h, respectively. An electroencephalogram (EEG), electrocardiogram, and heart rate were continuously monitored throughout the experiments.

A local anesthetic, lidocaine (Xylocaine; AstraZeneca, Osaka, Japan), was administered at pressure points and around surgical incisions. The depth of anesthesia was judged to be adequate because no significant heart rate change (>10%) was observed when the incision was made. The nictitating membrane was retracted and the pupil was dilated with topical application of tropicamide (0.5%), atropine (1%), and phenylephrine hydrochloride (0.5%) (Mydrin-P; Santen, Osaka, Japan). The eyes were refracted using contact lenses in order to focus them onto a cathode-ray tube (CRT) monitor. Body temperature was maintained at 38°C with a thermostatically controlled heating pad. The end-tidal CO_2_ concentration was adjusted to 4–5%.

Surgical procedures are described in detail elsewhere. A scalp was dissected after injecting lidocaine over the skull. For chiasmic stimulations, two openings were made in the skull, dura, and arachnoid near each side of the sagittal suture above and a pair of stimulation electrodes was inserted near each side of the optic chiasma (14.5 mm AP, 2.0 mm ML; Fukuda and Stone, 1974; Stone and Fukuda, 1974). After the recordings of an RGC, we stimulated the optic chiasma by passing a current (monophasic, a rectangular pulse of 50 μ s, 0.5–2 mA; Mihashi et al., 2011) and measured the response latency of the RGC for identification of the cell type (X, Y, or W) (see “Off-line Data Analysis” section).

For retinal recordings, a sclera was carefully stitched with a nylon suture to a fixation ring mounted on the stereotaxic head-holder. An opening was made in the sclera through which an intraocular guide tube was inserted and a tungsten electrode (FHC, USA; 3–5 MΩ) was inserted intraocularly through the guide tube (Takao et al., 2000, 2002). We carried the electrode forward while watching the fundus oculi with a funduscope and judged when the electrode contacted the retina by both the fundus image and the audio-monitoring of the spiking activity. For geniculate recordings, an opening was made in the skull, dura, and arachnoid above the LGN through which a tungsten electrode was vertically inserted (Naito et al., 2007; Suematsu et al., 2012).

### Visual stimulation, recordings, and on-line data analysis

Extracellular recordings were made from RGCs and LGN neurons using the tungsten electrodes. All stimuli were generated using custom-made MATLAB (Mathworks, USA) programs with Psychtoolbox (Brainard, 1997; Pelli, 1997) and presented on a gamma-corrected CRT monitor (FlexScan FX-E7, EIZO; mean luminance, 70 cd/m^2^; screen size, 40 × 30 cm^2^) placed 57 cm in front of the cat using two different settings (resolution 1280 × 960 pixels, refresh rate 85 Hz; or 1600 × 1200 pixels at 75 Hz). Electrophysiological signals were amplified using an AC amplifier (AM-1800; A-M Systems, USA) and sent to a slicer (Nihon Kohden, Tokyo, Japan), which performed on-line threshold-based spike detection. Digital pulses obtained from the slicer were acquired using an IO board (AIO-160802L-LPE, CONTEC, Osaka, Japan) and sampling rate of 20 kHz. Peristimulus time histograms (PSTHs) of the unit responses were constructed and analyzed off-line. We acquired the amplified raw signals for off-line spike sorting (see “Off-line Data Analysis”) and EEG signals to monitor an animal's vital conditions.

The center position of the RF was first assessed by carefully varying the spatial location of a small uniform patch stimulus. We then presented two-dimensional dynamic dense noise stimuli (size, 9.6 × 9.6 or 16 × 16° divided into 31 × 31 or 61 × 61 grids; duration = 10 min) every two frames of the monitor refresh at the assessed position on the CRT monitor (median spike rates of RGCs and LGN neurons = 97 and 78 spk/s, respectively), and the spatiotemporal RF structure was reconstructed using the reverse correlation technique (Jones and Palmer, 1987).

### Off-line data analysis

To conduct off-line spike sorting, we used Wave_Clus (Quiroga et al., 2004) running on MATLAB. Raw data were band-pass filtered, spikes were detected from the filtered data on the basis of a particular threshold (usually threefold baseline noise level calculated from absolute values of the filtered data), features of the detected spikes were extracted with the wavelet analysis, and the detected spikes were clustered into multiple single units on the basis of the extracted features. We confirmed the existence of 3-ms or more refractory period in the auto-correlogram of spike trains for all neurons.

**Figure 1:**
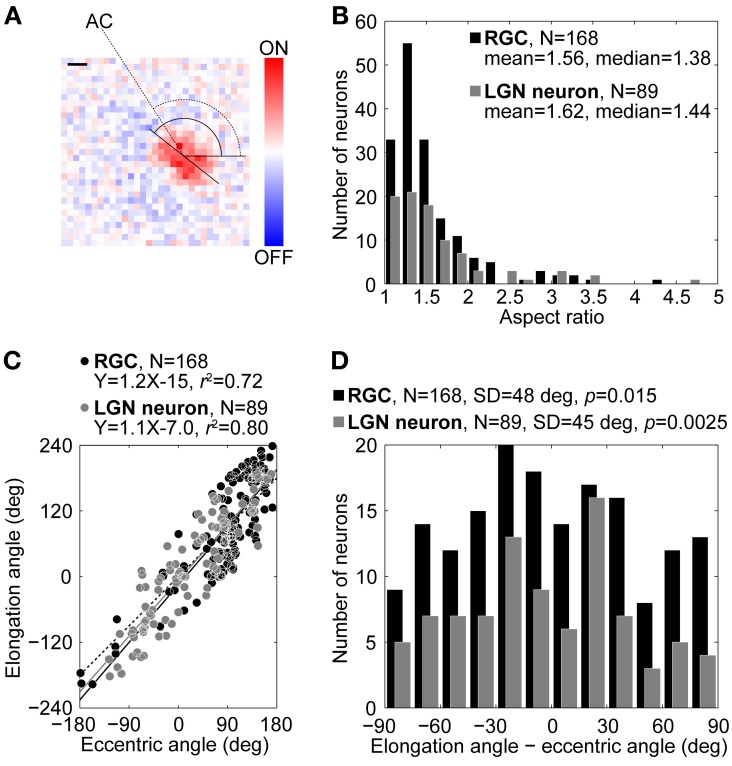
**Comparison of RF structures of RGCs and LGN neurons. (A)** Example of a RF structure of an ON-center RGC at the peak response latency (40 ms). Reddish and bluish colors indicate ON- and OFF-responses, respectively. The aspect ratio, elongation angle (solid arc) of the center region, and eccentric angle (dotted arc) were 1.38, 142, and 123°, respectively. AC indicates the *area centralis*. Scale bar = 1°. **(B–D)** Distributions of the aspect ratios **(B)**, relationship between the elongation and eccentric angles (see details in text) **(C)**, and distributions of differences between elongation and eccentric angles **(D)**. Black and gray bars/circles indicate RGCs and LGN neurons, respectively.

RGCs were classified as X-, Y-, or W-cells based on the second to first harmonic (F2/F1) ratio of the response, response latency, and RF size (Enroth-Cugell and Robson, 1966; Stone and Fukuda, 1974; Hochstein and Shapley, 1976), while LGN neurons were classified as X- or Y-cells based on the F2/F1 ratio only (Bonin et al., 2005). A spatiotemporal RF structure was reconstructed from single-unit activity (typically 3000–25000 spikes) and the reverse correlation technique. We fitted the reconstructed spatial RF structures at the peak response latency with the two-dimensional difference of Gaussians (2DDoG) model, as previously described (Suematsu et al., 2012). This approach provided the center position, aspect ratio, elongation angle (angle between the long axis of the RF and horizontal meridian, solid arc in Figure 1A), and size (2SD of the fitted center Gaussian) of the spatial RF structures. In addition, we calculated the eccentric angle, angle between the horizontal meridian and the line connecting the RF center position with the *area centralis* (dotted arc in Figure 1A). Elongation angles and eccentric angles essentially ran from −180 to 180°. We chose elongation angles from cells so that the difference in eccentric and elongation angles became acute. Note that the population of eccentric angle data distributed in the range between 0 and 180°, especially for RGCs (RGC, *N* for eccentric angle < 0 and ≥ 0 were 15 and 153, respectively; LGN neurons, *N* = 40 and 49, respectively). This is because the number of recoded RGCs was larger at the ventral side of the *area centralis* than that at the dorsal side.

The cross-correlogram (XC) was calculated from the single-unit activity elicited by noise stimuli using the following equation (Usrey et al., 1999) to assess monosynaptic retinogeniculate connections:

(1) $$XC(\tau )=\Sigma_{t}R_{i}(t)R_{j}(t+\tau )$$

where *R*_*i*_*(t)* indicates the response (spikes/s) of the *i*-th single unit at the *t*-th time bin (width = 0.1 ms). Indices *i* and *j* indicate retinal and geniculate single units, respectively. We filtered the raw *XC* with a band-pass filter whose frequency characteristic was Gaussian shaped with mean = 1 kHz and variance = 707 Hz. These values were determined to make the frequency properties analogous to the filter used in a previous study (Usrey et al., 1999). To estimate the baseline noise level, we calculated the mean and *SD* of the filtered *XC* between ±10 ms after removing the 2–5 ms interval. We defined a significant retinogeniculate connection when the filtered *XC* in the 2–5 ms interval exceeded the baseline +5 *SD*. Also we calculated the efficacy (peak *XC* amplitude normalized by the number of retinal spikes) and contribution (peak *XC* amplitude normalized by the number of geniculate spikes) as the measure of the connection strength (Usrey et al., 1999).

To quantitatively evaluate a spatial relationship between structures, including distance between the RF center positions, and each RF size, the overlap ratio was calculated from the spatial RF structures of a retinogeniculate pair that had monosynaptic connections using the following equation:

(2) $$\text{overlap ratio}=(S_{i}+S_{j})/D$$

where *S* and *D* indicate the RF size and the distance between the RF center positions of an RGC and its connected LGN neuron, respectively. More specifically, *S* was defined as the distance from the RF center position to the intersection with an ellipse approximating the RF (2 *SD* of the center Gaussian) along line *D* (see Figure 5C, inset). Overlap ratios greater than 1 indicate that center regions of the spatial RF structures of the connected pair are overlapped, while those less than 1 indicate they are not. This measure contains the inter-RF-centers distance and RF sizes of the RGC and LGN neuron, and thus is assumed as the distance normalized by the RF sizes.

Singular value decomposition (SVD) of the spatiotemporal RF structure was performed to extract the temporal RF structure. SVD allows us to decompose a spatiotemporal RF structure into three components: a spatial RF structure, a temporal RF structure (two eigenvector matrices), and an amplitude (an eigenvalue matrix) (Wolfe and Palmer, 1998). In practice, to conduct SVD, we used the MATLAB command svd after reshaping a 3D spatiotemporal RF structure (space × space × time) into 2D (space × time). The separability of spatial and temporal RF structures was confirmed by the calculating the percentage of total power captured by the first eigenvalue. For our data, this measure ran between 20 and 72% for the RGCs and between 8 and 54% for the LGN neurons, whose ranges were somewhat lower than a previous study (Wolfe and Palmer, 1998, 36–90%). This difference was probably caused by the resolution of the RF structures; we used two-dimensional noise stimuli which have total 961 (= 31 × 31) or 3721 (= 61 × 61) positions, whereas they used one-dimensional 16-position bar stimuli. To compare the temporal RF structure between each retinogeniculate pair, we first normalized the intensities by the first response peak value and then extracted the peak latency of the first response (primary peak latency, P1), the peak latency of the rebound response (secondary peak latency, P2), the duration of the first response (full width at half maximum, FWHM1), the duration of the rebound response (full width at half minimum, FWHM2), and the relative amplitude of the rebound response to first response (minimum intensity, *m*) (Figure 8).

### Histology of the LGN

At the end of each penetration for the LGN recordings, at least three electrolytic lesions were made along the track by passing a current (DC, 3–4 μ A, 10 s, tip negative). Lesions were separated by intervals of more than 300 μm. After the recording experiments, the animals were deeply anesthetized with sodium pentobarbital (60 mg/kg, i.v.) and perfused transcardially with phosphate buffered saline (PBS, pH 7.4). Blocks of the dorsolateral thalamus were obtained and immersed in 30% sucrose in 4% paraformaldehyde for 36–48 h. Frozen sagittal sections 80-μ m thick were sliced on a microtome and kept in PBS. Sections were stained for cytochrome oxidase or Nissl substance. The laminar locations of the recording sites were then identified under a light microscope. Shrinkage in the geniculate tissues was corrected for by multiplying the ratio of the measured lesion distance by the value calculated from the micrometer reading. LGN layers were classified as layers A, A1 or C.

## Results

We obtained the single-unit activities of 168 RGCs (*X, Y, W* = 136, 31, 1) and 34 LGN neurons (Layer A, A1, *C* = 9, 4, 11; *X*, *Y*, unknown = 12, 1, 21). For all 26 retinogeniculate pairs monosynapticaly connected, as confirmed by the *XC*, cell types and their combinations are summarized in Table 1. RF positions of the RGCs were not confined to a particular retinal location (from nasal 30° to temporal 14°, from ventral 33° to dorsal 13°). Among the 105 retinogeniculate pairs analyzed, 26 pairs exhibited significant retinogeniculate connections according to cross-correlation analysis. We compared the spatiotemporal RF structures of the RGCs (*N* = 168) and the LGN neurons (*N* = 89; 34 neurons recorded in the current study and 55 neurons recorded in our previous study, Suematsu et al., 2012).

**Table 1:** **Cell types for the retinogeniculate pairs**.

		**LGN neuron**			
		***X***	***Y***	**Unknown**	
**RGC**	***X***	**7**	**3**	**11**	**21**
	***Y***	**2**	**0**	**3**	**5**
	***W***	**0**	**0**	**0**	**0**
		**9**	**3**	**14**	**26**

### Comparison of spatial rf structures of RGCs and LGN neurons

Figure 1A shows a representative example of the spatial RF structure of an X-type RGC at the peak response latency (40 ms). The spatial RF structure was elliptically elongated ON-center and OFF-surround. For most pairs, the surround structures were so obscure that we did not analyze them parametrically. The aspect ratio, elongation angle, and eccentric angle were 1.38, 142, and 123°, respectively. Overall, we focused on these three parameters for our analysis of all RGCs and LGN neurons and their comparisons.

Figure 1B shows the distributions of the aspect ratio of the spatial RF structures. Black and gray bars indicate RGCs (*N* = 168, mean = 1.56, median = 1.38) and LGN neurons (*N* = 89, mean = 1.62, median = 1.44), respectively. The aspect ratio of the spatial RF structure of the RGCs was not significantly different from that of the LGN neurons (two-sample Kolmogorov–Smirnov test, *p* = 0.64), indicating that the RGCs had RFs as elongated as those of the LGN neurons.

Figure 1C shows a relationship between the elongation angles and eccentric angles. Black and gray circles indicate RGCs and LGN neurons, respectively. For both RGCs and LGN neurons, most data points were distributed around the diagonal line (dotted line). The regression lines calculated with the least-squares method are *Y* = 1.2X–15 (coefficient of determination, *R*^2^ = 0.72) for the RGCs (solid line) and *Y* = 1.1X–7.0 (*R*^2^ = 0.80) for the LGN neurons (gray solid line). These results suggest that the RF structure of both the RGCs and LGN neurons were elongated in a direction toward the *area centralis*. To verify this possibility in detail, we calculated differences between the elongation angles and the eccentric angles (Figure 1D). For both the RGCs and the LGN neurons, the distributions were significantly different from a uniform distribution and were biased to 0° (RGC, *SD* = 48°, *v*-test, *p* = 0.015; LGN neuron, *SD* = 45°, *p* = 0.0025), further suggesting that the spatial RF structures of both the RGCs and the LGN neurons tended to be oriented in a direction toward the *area centralis*. There was no significant difference between the two distributions (two-sample Kolmogorov–Smirnov test, *p* = 0.70).

These results support the possibility that the elongated RF structure of an LGN neuron is derived from that of its input-source RGC. To assess this possibility directly, we compared the spatial RF structures of pairs with retinogeniculate connections.

### Relationship of spatial RF structures of connected pairs

**Figure 2:**
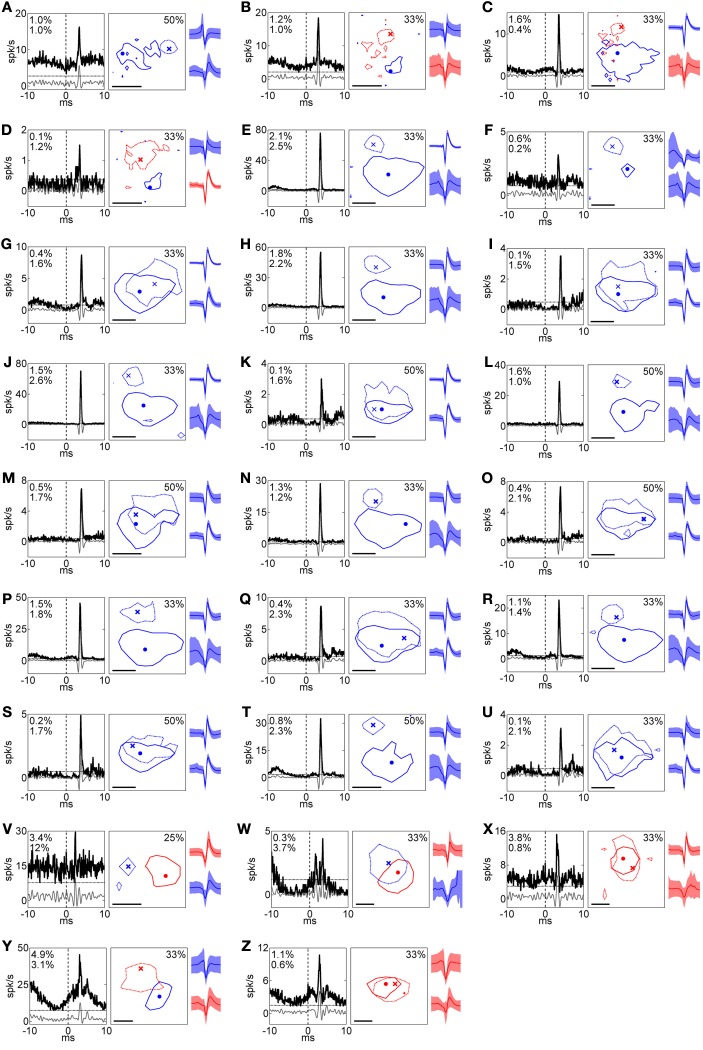
**Summary of RFs and corresponding *XC*s for all pairs. (A)–(Z)** Left, middle, and right columns indicate *XC*, RF, and spike shape, respectively. In the *XC* columns, black solid, gray solid, and horizontal dotted lines indicate raw *XC*, filtered *XC*, and threshold (mean + 5 *SD*), respectively, and numbers read efficacy (upper) and contribution (lower). In RF columns, solid and dotted lines indicate the RFs of the RGC and the LGN neuron, respectively. Numbers read the response levels of the contour lines for the both of units. Dot and cross symbols indicate the maximum response positions of the RGC and the LGN neuron, respectively. Scale bar = 1°. In spike shape columns, solid lines and shaded areas indicate mean and 1 *SD*, respectively. Colors correspond to the response sign (red, ON; blue, OFF). Upper and lower shapes are for an RGC and LGN neuron, respectively. Pairs in Figures 3, 4 correspond to **(S)** and **(C)**, respectively. **(B)** and **(C)** are reconstructed from the same recording and exhibited the same LGN neuron with different RGCs, indicating two OFF-center RGCs were projecting to one ON-center LGN neuron.

**Figure 3:**
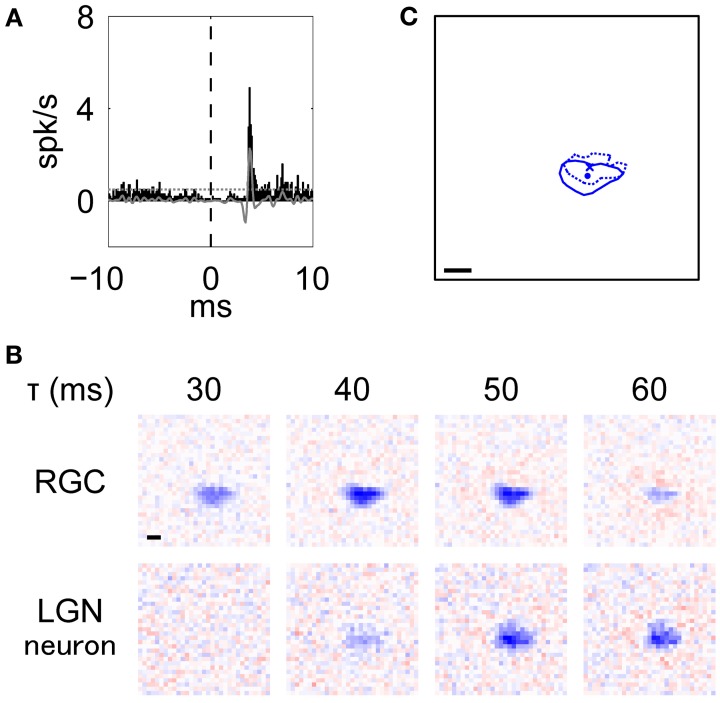
**Typical example of an RGC-LGN neuron pair with RFs of the same response sign.** This was the pair shown in Figure 2S. **(A)** *XC* of the pair. Black bars and gray line indicate raw and filtered data, respectively. Horizontal dotted line indicates mean + 5 *SD*. **(B)** Spatiotemporal RF structures (top: RGC, bottom: LGN neuron; left to right: shorter to longer latencies). **(C)** Overlaid image of the RF centers. Solid line and dot indicate 50% of response level and center position of the RGC, respectively. Dotted line and cross-symbol indicate those of the LGN neuron. The RF center positions were obtained from the fitted parameters. In **(B)** and **(C)**, scale bar = 1°.

**Figure 4:**
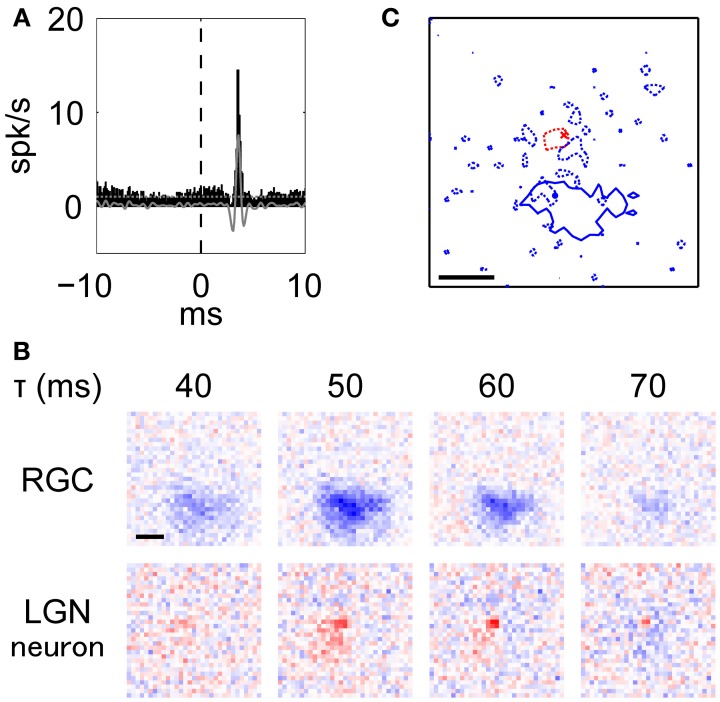
**Typical example of an RGN-LGN neuron pair with RFs of the opposite response sign.** This was the pair shown in Figure 2C. **(A)–(C)** Details are the same as Figure 3. Note that the blue dotted lines in **(C)** indicate the RF surround of the LGN neuron at 25% response level at latency 70 ms.

We identified 26 pairs of single units with electrophysiologically assessed (see Methods, Equation 1) retinogeniculate connections from simultaneously recorded RGCs and LGN neurons (Figure 2). We found that there were two types of retinogeniculate connections between an RGC-LGN neuron pair: those that exhibited RFs with the same response sign (20/26) and those that exhibited RFs with the opposite response sign (6/26). Figures 3, 4 show typical examples of same- and opposite-response-sign pairs, respectively.

Figure 3A shows an example of an *XC* (see Methods, Equation 1) with a peak at 3.8 ms, indicating that there was a monosynaptic connection between the RGC and the LGN neuron. Figure 3B shows the spatiotemporal RF structures of this pair (top, RGC; bottom, LGN neuron). The RGC exhibited a horizontally-elongated OFF-center RF structure (aspect ratio and elongation angle = 1.95 and −3°, respectively), which is similar to that of the target LGN neuron (aspect ratio and elongation angle = 1.65 and 0°, respectively) and precedes it by about 10 ms. Note that although there were large separations between 3.8 ms from the *XC* and 10 ms from the RFs, we attribute this to stimuli being refreshed at 75 or 85 Hz (see Methods; one-frame duration = 13 or 12 ms). The overlaid RF structures at each peak response latency (RGC, 44 ms; LGN neuron, 55 ms) shown in Figure 3C (Pearson product-moment correlation coefficient, *r* = 0.60, *t*-test of a correlation coefficient, *p* = 8.7 × 10^−97^) further suggests that the elongated RF structure of LGN neurons directly reflects that of their projecting RGCs.

There was a small population of retinogeniculate pairs with monosynaptic connections that had opposite signs for the RF center. Figure 4A shows an example of an *XC* with a peak at 3.6 ms, indicating a monosynaptic connection between the RGC and the LGN neuron. Figure 4B shows the spatiotemporal RF structures of the pair (top, RGC; bottom, LGN neuron). This RGC exhibited a horizontally-elongated OFF-center RF structure (aspect ratio and elongation angle = 1.44 and 174°, respectively), while the target LGN neuron exhibited an ON-center RF structure (aspect ratio and elongation angle = 1.18 and 180°, respectively). Figure 4C shows overlaid images of the RFs shown in Figure 3B at each peak response latency (RGC, 47 ms; LGN neuron, 57 ms). These two spatial RF centers were not overlapped, rather the OFF-center RGC seemed to partially overlap the OFF-surround region of the LGN neuron (*r* = 0.18, *t*-test of a correlation coefficient, *p* = 5.7 × 10^−27^), suggesting that this RGC contributed to generating the antagonistic surround region of the target LGN neuron.

These above results suggest that there are two types of retinogeniculate connections; one with the same response sign, which probably generates the RF center of the LGN neuron, and the other with the opposite response sign which, may correspond to the RF surround. To examine this hypothesis in detail, we compared the RF properties (difference of elongation angles, distance between RF center positions, overlap ratio, and *r* between spatial RF structures) between each retinogeniculate pair.

### Comparison between same- and opposite-response-sign pairs

**Figure 5:**
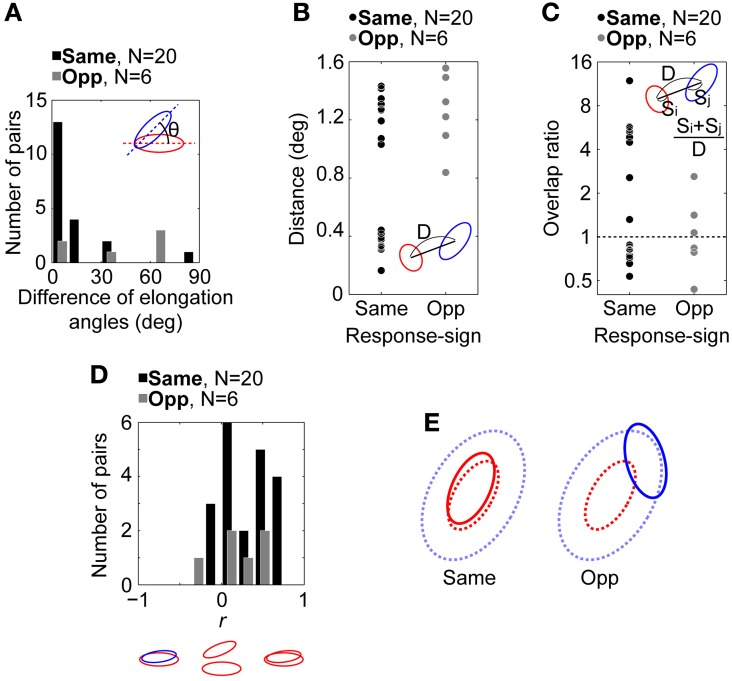
**Relationship between the spatial RF structures of retinogeniculate-connected pairs (*N* = 26). (A)** Distributions of the difference of elongation angles of the spatial RF structures. **(B)** Distributions of RF center distances of the pairs. **(C)** Distributions of the overlap ratios between the antagonistic RF center regions of the pairs. **(D)** Distributions of *r* between the spatial RF structures of the pairs. An RGC-LGN neuron pair exhibit completely overlapped RF structures with the same response sign when *r* = 1 and completely overlapped RF structures with the opposite response sign when *r* = −1. In **(A–D)**, insets indicate schematic spatial RF structures. θ, D, S indicate the difference of elongation angles, distance between RF center positions, and size of spatial RF structure, respectively. Black and gray bars/circles indicate pairs with RFs of the same (*N* = 20) and opposite response sign (*N* = 6), respectively. **(E)** Schematic summary of spatial RF structures in the retinogeniculate connections. Solid and dotted lines indicate spatial RF structures of RGCs and the corresponding target LGN neurons, respectively. In same- and opposite-response-sign pairs, RGCs exhibit ON-center and OFF-center RFs, respectively. In both pairs, LGN neuron exhibits ON-center OFF-surround RF.

Figure 5A shows the distribution of the difference of elongation angles between the spatial RF structures of connected pairs. A majority of pairs with the same response sign (17/20, black bars) were distributed within a difference of 0–20°, while data for pairs with the opposite response sign were more evenly distributed (*N* = 6, gray bars). There was a significant difference between the medians of these two distributions (5.2 and 50.4°, respectively; Wilcoxon rank sum test, *p* = 0.019; bootstrap test based on Wilcoxon rank sum test statistic, *n* = 10000, *p* = 0.0028). These results indicate that the same-response-sign pairs exhibited similar oriented RF structures, while the opposite-response-sign pairs did not.

Figure 5B shows the distributions of the distances between the RF center positions of the pairs. For the same-response-sign pairs (left, black dots), data points were distributed widely, ranging from 0.16 to 1.43° (mean and median = 0.86 and 1.05°, respectively). On the other hand, the opposite-response-sign pairs (right, gray dots) tended to exhibit relatively longer distances, ranging from 0.84 to 1.56° (mean and median = 1.25 and 1.27°, respectively, Wilcoxon rank sum test, *p* = 0.09), suggesting that for these pairs, the RF center region of the RGCs overlapped with the RF surround region of the target LGN neurons.

Because, however, the RF sizes are different from each other, we calculated the overlap ratio as the distance normalized by the RF sizes of the pairs. Figure 5C shows the distributions of the overlap ratios (see Methods, Equation 2) between the RF center regions of all pairs. Half of the same- (10/20) and opposite- (3/6) response-sign pairs had overlap ratios greater than 1, indicating their RFs overlapped each other's center region, whereas the other half had overlap ratios smaller than 1, indicating those RFs did not overlap. The same-response-sign pairs exhibited a wide range of overlap ratios (0.53–12, geometric mean = 1.9), while the opposite-response-sign pairs had a significantly smaller range (0.44–2.6, geometric mean = 1.0; *t*-test after logarithmic transformation, *p* = 0.046). This result indicates that the RGCs in the same-response-sign pairs tended to exhibit spatial RF structures that overlap the LGN neurons' RF center and surround regions, whereas those in the opposite-response-sign pairs exhibited RFs overlapping only the surround region of the RFs of the LGN neurons.

Figure 5D shows the distributions of *r* between the spatial RF structures of different pairs. Most data are distributed on the positive side of the abscissa, indicating that the RF regions, be they center or surround, can overlap if their response signs are the same. In other words, ON-center RGCs overlap the ON-center region of ON-center LGN neurons (same-response-sign pairs) or the ON-surround region of OFF-center LGN neurons (opposite-response-sign pairs) and vice versa.

It could be argued that in the same-response-sign pairs of Figure 5B there exist two clusters (left, black dots), one with relatively small differences in the RF center positions (SHORT, distance < 0.8°, *N* = 9), in which the RFs of the pairs can overlap each other's center region, and one with relatively large differences (LONG, distance ≥ 0.8°, *N* = 11), in which the RF center region of the RGCs probably overlap the RF surround region of the target LGN neurons rather than the center. We compared other spatial RF properties (difference of elongation angles, overlap ratio, and *r*) between these two groups, finding significant differences in the overlap ratio and *r* (overlap ratio, geometric mean for SHORT and LONG = 5.2 and 0.80, respectively, median = 5.3 and 0.82, respectively, Wilcoxon rank sum test, *p* = 2.0 × 10^−4^; *r*, mean = 0.56 and 0.098, respectively, median = 0.59 and 0.034, respectively, Wilcoxon rank sum test, *p* = 6.3 × 10^−4^), but not in the elongation angles (mean for SHORT and LONG = 12 and 13, respectively, median = 3 and 13, respectively, Wilcoxon rank sum test, *p* = 0.25). These results are as expected, since the longer distance makes a smaller overlap ratio (see Equation 2) and the LONG pairs have RFs with the same response sign at their center positions. Thus, the two clusters show differences just in their RF center distances, and any functional differences, such as displaced inputs being orientation-independent, are not suggested. However, because the number of connected pairs is small, future studies are necessary to validate these conclusions.

Taken together, it is suggested that a single LGN neuron receives two types of convergent inputs from RGCs, one which exhibits the same response sign and a similarly oriented RF to primarily determine the RF center of the target LGN neuron, and another which exhibits the opposite response sign and an independently oriented RF to primarily determine the antagonistic RF surround (Figure 5E).

**Figure 6:**
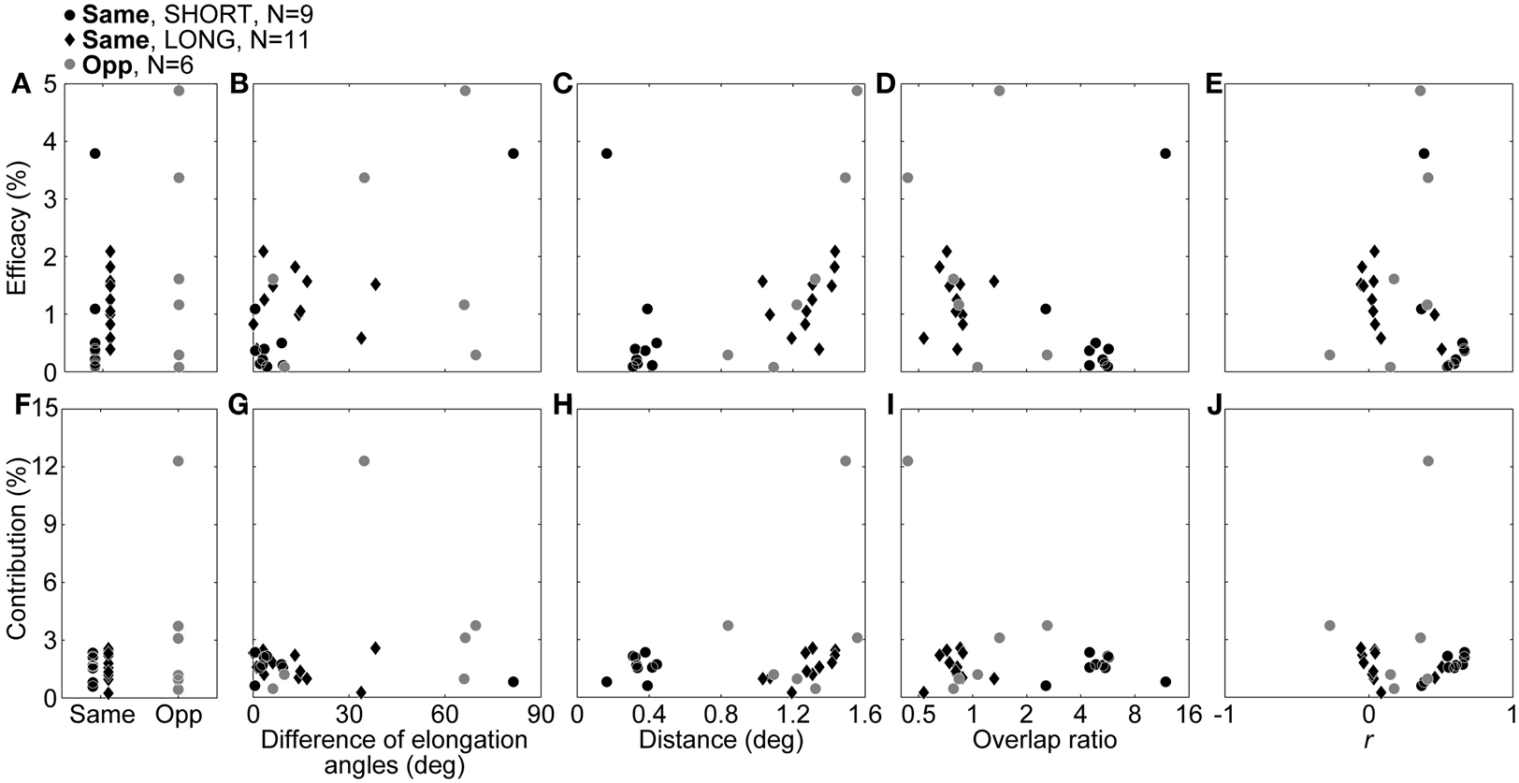
**Efficacy and contribution, and the correlations with RF properties.** In the upper row, the efficacy itself **(A)**, correlations with the difference of the elongation angles **(B)**, distance between RF center positions **(C)**, overlap ratio **(D)**, and correlation coefficient of the RF structures **(E)**, are shown. In the lower row, the contribution itself **(F)**, correlations with differences of elongation angles **(G)**, distance between RF center positions **(H)**, overlap ratio **(I)**, and correlation coefficient of the RF structures **(J)** are shown. Black dots, black diamonds, and gray dots indicate SHORT, LONG, and opposite-response-sign connections, respectively.

Next, we compared connection strength between same- and opposite-response-sign pairs. To this aim, we compared the efficacies and contributions (see Methods) between the same- and opposite-response-sign pairs (Figures 6A,F). We also investigated the relationships between the efficacies/contributions and the RF properties of the pairs (Figures 6B–E, G–J). We used Spearman's rank correlation coefficient, which is a nonparametric measure of statistical correlation, instead of Pearson product-moment correlation coefficient for the comparisons, because there seemed to be outliers (Dixon's test, *p* for the efficacy in the same sign and the contribution in the opposite sign = 0.0091 and 0.0071, respectively) which may lead to artificial correlations.

Figure 6A shows comparisons of the efficacies between same- and opposite-response-sign pairs. There was no significant difference in the efficacy between the same- and the opposite-response-sign connections (median efficacy for same- and opposite-response-sign connections = 0.91 and 1.39, respectively; Wilcoxon rank sum test, *p* = 0.45), suggesting that the opposite-response-sign inputs were similarly efficient to the same-response-sign inputs. However, contrary to expectation, SHORT had an efficacy significantly smaller than LONG (median efficacy for SHORT and LONG = 0.37 and 1.25, respectively; Wilcoxon rank sum test, *p* = 0.019), indicating that the displaced inputs were more efficient. Furthermore, there was a significant negative correlation between the efficacy and the correlation coefficient of the RF structures in LONG (Figure 6E, black diamonds, ρ = −0.67, *p* = 0.028). These results suggest that, in LONG, connections with higher efficacies have less similar RF structures. Similarly, in the opposite sign, there was a significant positive correlation between the efficacy and the inter-RF-centers distance (Figure 6C, gray dots, ρ = 0.94, *p* = 0.017), indicating that connections with higher efficacy share displaced RFs. In the remaining cases, no significant correlations were observed.

For the contribution, there were no significant differences between the response signs (Figure 6F, black symbols vs. gray dots, median contributions for the same and opposite signs = 1.62 and 2.14, respectively, Wilcoxon rank sum test, *p* = 0.61) or between SHORT and LONG (Figure 6F, black dots vs. black diamonds, median contributions for SHORT and LONG = 1.66 and 1.57, respectively, Wilcoxon rank sum test, *p* = 0.88). In SHORT, as expected, the contribution had a significant positive correlation with the correlation coefficient of the RF structures (Figure 6J, black dots, ρ = 0.73, *p* = 0.031), indicating that those with higher contribution share similar RFs. In LONG, there was a significant positive correlation between the contribution and the inter-RF-centers distance (Figure 6H, black diamonds, ρ = 0.71, *p* = 0.019). Again, the LONG connections with higher connection strength have less similar RF structures.

**Figure 7:**
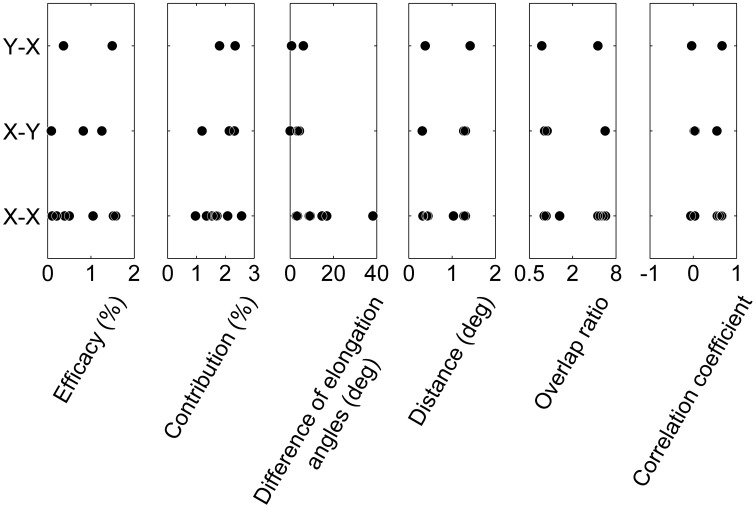
**Relationships between cell types and RF properties.** From left to right, the relationships between the cell types in connection (*X*–*X*, *X*–*Y*, *and Y*–*X*) and efficacy, contribution, difference of elongation angles, inter-RF-centers distance, overlap ratio, and correlation coefficient of RF structures are shown. Note that the range of some abscissae is different to those in Figures 5, 6 for ease of viewing.

In addition, we investigated the relationships between the cell types of pairs (X RGC-X LGN neuron, *X*–*Y*, and *Y*–*X*; Table 1) and other measures (efficacy, contribution, difference in elongation angles, inter-RF-centers distance, overlap ratio, and correlation coefficient of RF structures). There were no significant differences in the measures among cell types of the pairs (Figure 7; efficacy, Kruskal-Wallis test, χ^2^_(2, 9)_ = 0.22, *p* = 0.89; contribution, χ^2^_(2, 9)_ = 1.48, *p* = 0.48; difference of elongation angles, χ^2^_(2, 9)_ = 3.51, *p* = 0.17; inter-RF-centers distance, χ^2^_(2, 9)_ = 0.42, *p* = 0.81; overlap ratio, χ^2^_(2, 9)_ = 0.84, *p* = 0.66; correlation coefficient of RF structures, χ^2^_(2, 9)_ = 0.42, *p* = 0.81). However, the populations seem too small (*N* for *X*–*X*, *X*–*Y*, *and Y*–*X* = 7, 3, and 2, respectively) to draw conclusive remarks.

### Comparison of temporal RF structures of connected pairs

In the previous section, we found that the spatial RF structure of LGN neurons derives from two types of convergent inputs from RGCs. In this section, to clarify the underlying mechanisms for the generation of the temporal RF structure from the two types of convergent inputs, we calculated the temporal RF structures using SVD (see Methods), and then compared the structures between each retinogeniculate pair.

**Figure 8:**
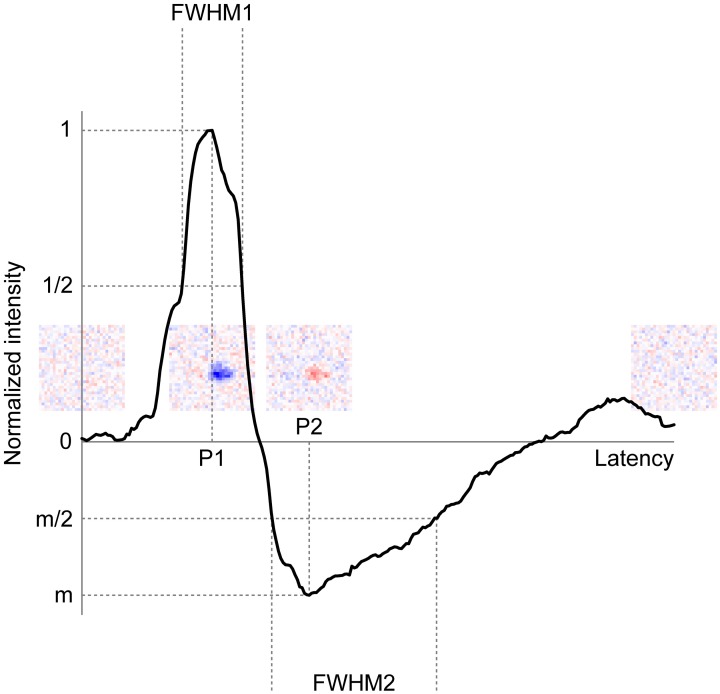
**Typical example of the temporal RF structure of LGN neurons.** Horizontal and vertical axes indicate latency and normalized intensity of the temporal RF structure, respectively. P1, P2, FWHM1, FWHM2, and m indicate peak latency of the first response, peak latency of the rebound response, duration of the first response, duration of the rebound response, and relative amplitude of the rebound response, respectively. Insets indicate spatial RF structures at latencies of 0, 55 (P1), 96 (P2), 250 ms, respectively.

Figure 8 shows a representative example of a temporal RF structure of the LGN neuron shown in Figure 3. This neuron showed a strong OFF-response (see Figure 8, inset second from left) with a short latency (first response), and then a weak ON-response (see Figure 8, inset third from left) with a long latency (rebound). From the temporal RF structure, we extracted the peak latency of the first response (P1), peak latency of the rebound response (P2), duration of the first response (FWHM1), duration of the rebound response (FWHM2), and the relative amplitude of the rebound response to the first response (*m*). For example, for the LGN neuron in Figure 8, P1, P2, FWHM1, FWHM2, and m were 55, 96, 27, 70 ms, and −0.49, respectively. We then compared these values between the two cells of each retinogeniculate-connected pair to examine the possibility that the temporal RF structure of the LGN neurons is derived from the two types of convergent inputs from the RGCs.

**Figure 9:**
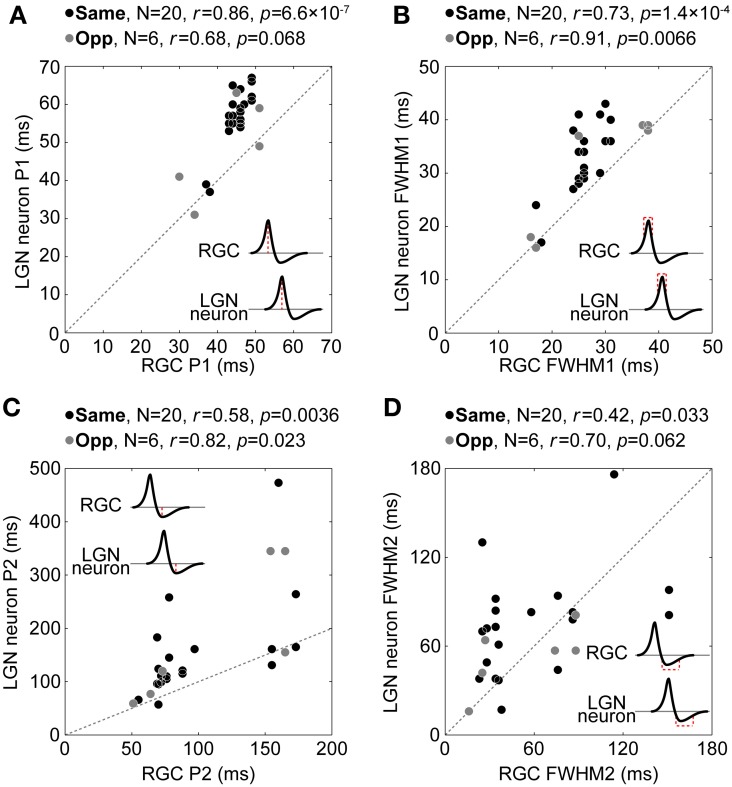
**Relationship between temporal RF structures of retinogeniculate-connected pairs.** Relationship between P1 **(A)**, FWHM1 **(B)**, P2 **(C)**, and FWHM2 **(D)** for RGCs (horizontal axis) and LGN neurons (vertical axis). Black and gray circles indicate same- and opposite-response-sign pairs, respectively. Insets indicate schematic temporal RF structures. Note that the scale of the horizontal axis and that of the vertical axis are not equal in **(C)**.

Figure 9 shows comparisons of P1 (Figure 9A), FWHM1 (Figure 9B), P2 (Figure 9C), and FWHM2 (Figure 9D) between each pair. There were significant positive correlations or similar tendencies between each temporal property (P1, *r* for same and opposite response signs = 0.86 and 0.68, respectively, *t*-test of a correlation coefficient, *p* = 6.6 × 10^−7^ and = 0.068; FWHM1, *r* = 0.73 and 0.91, *p* = 1.4 × 10^−4^ and = 0.0066; P2, *r* = 0.58 and 0.82, *p* = 0.0036 and = 0.023; FWHM2, *r* = 0.42 and 0.70, *p* = 0.033 and = 0.062). These tendencies disappeared with the randomly sampled pairs (repeat count = 10000, *r* for P1, mean ± 1.96 × s.e.m. = −0.0030 ± 0.0039; FWHM1, −9.3 × 10^−4^ ± 0.0039; P2, −0.0017 ± 0.0039; FWHM2, −0.0019 ± 0.0039), indicating that a projecting RGC and its target LGN neuron exhibit similar temporal RF structures. These results suggest that the temporal RF structure of LGN neurons derives from the convergent inputs of two types of RGCs that have the same or opposite response sign of the spatial RF over different time courses.

Figure 9A also shows pairs in which a target LGN neuron has P1 equal to or shorter than that of its projecting RGC. In addition, in Figure 9B, LGN neurons show significantly longer FWHM1 than their projecting RGCs (medians for RGC and LGN neuron = 26 and 35, respectively, Wilcoxon signed-rank test, *p* = 0.0016). These results support the idea that there exist convergent retinogeniculate connections of various input latencies; the sum of multiple inputs of various latencies have a temporal range wider than the inputs (longer FWHM1), and in these cases, the latency of the sum is shorter for later inputs (shorter P1).

## Discussion

In the current study, we simultaneously recorded the single-unit responses of RGCs and LGN neurons in cat and compared the spatiotemporal RF structures between neuron pairs monosynaptically connected. The results are summarized as follows: (1) RGCs exhibited elliptically elongated RF structures oriented in a direction toward the *area centralis*, and their aspect ratios (mean = 1.56, *N* = 168) were comparable to those of LGN neurons (mean = 1.62, *N* = 89); (2) for monosynaptically connected retinogeniculate pairs with RFs of the same response sign, the center regions of the two RFs were overlapped and oriented similarly; (3) for pairs with RFs of the opposite response sign, the center regions of the two RFs were spatially displaced and oriented independently; (4) for both populations of connected pairs, the RF spatial properties seemed to have relationships with the connection strength (efficacy and contribution); and (5) for both the populations, temporal RF structures were tightly correlated and the LGN neurons had significantly longer response durations than the RGCs. These results suggest that the spatiotemporal RF structure of a cat LGN neuron is mainly inherited from the dominant inputs of a projecting RGC, while convergent inputs from multiple RGCs may be responsible for enhancing its antagonistic center and surround regions.

### Spatial RF structure of RGCs

We found that RGCs exhibit an elliptical RF structure that is comparable to that seen in LGN neurons (Figures 1A,B). We also found that the spatial RF structures of the RGCs and LGN neurons were oriented in a direction toward the *area centralis* (Figures 1C,D).

Our current results are consistent with previous studies reporting an elliptical spatial RF structure of cat RGCs (Rodieck and Stone, 1965; Hammond, 1974) and directional characteristic of RF elongation of cat LGN neurons (Vidyasagar and Urbas, 1982). Yet how the elongated RF structure of RGCs is generated remains unclear. One possible explanation is that the RF structure reflects an anisotropic dendritic arborization of the RGCs. In fact, previous studies have reported that the dendritic fields of cat RGCs are elliptical (Boycott and Wässle, 1974; Leventhal and Schall, 1983) and oriented radially (Leventhal and Schall, 1983). Similarly, in primate, it has been reported that RGCs exhibit orientation selectivity (Passaglia et al., 2002) or radially-oriented dendritic field structures (Rodieck et al., 1985; Schall et al., 1986; Watanabe and Rodieck, 1989; Szmajda et al., 2005). On the other hand, in tree shrew, it is generally thought that orientation selective neurons first emerge in the visual cortex, especially layer 2/3 (Fitzpatrick, 1996; Bosking et al., 1997; Chisum et al., 2003; Scholl et al., 2013; Van Hooser et al., 2013; Veit et al., 2013), while only few neurons in the retina exhibit orientation selectivity (Van Dongen et al., 1976; 6/93 neurons they recorded). Thus, these results suggest that the elliptically elongated RF structure of RGCs is an essential property despite species differences.

### Retinogeniculate connections and response-signs

We found that the majority of retinogeniculate-connected pairs exhibit same-response-sign RF structures (Figures 2, 3, 5, 20/26 pairs), consistent with a previous study (Usrey et al., 1999). To our knowledge, there is little evidence that OFF- (ON-) center RGCs project to ON- (OFF-) center LGN neurons. Usrey et al. (1999) investigated the preference of the retinogeniculate connections in cat, reporting that one of twelve pairs had an opposite-response-sign RF. In the current study, we found six such pairs out of 26 pairs with retinogeniculate connections (Figures 2, 4, 5). Thus, it is clear that LGN neurons receive both opposite-response-sign and same-response-sign inputs from RGCs.

Our results strongly suggest that the opposite-response-sign inputs from the RGCs contribute to responses in the antagonistic RF surround region of the target LGN neurons. A similar model has been proposed by Hammond (1973) that describes a single LGN neuron receiving one same-response-sign input and multiple opposite-response inputs, which correspond to the antagonistic center and surround region of the LGN neuron, respectively, from RGCs. The opposite-response-sign inputs may enhance the center-surround antagonism of the RF of the target LGN neurons compared to that of the projecting RGCs.

Neurons having spatial RF structures with stronger antagonism will exhibit more band-pass SF selectivity, because the center region has low-pass selectivity with a higher cut-off and the antagonistic surround region, which has low-pass selectivity with a lower cut-off, reduces responses to low-band SF stimuli. Cheng et al. (1995) investigated the SF selectivity of LGN neurons and projecting RGCs by recording S-potentials in cat LGN, demonstrating that the RGCs exhibited low-pass SF selectivity, while the target LGN neurons exhibited more band-pass SF selectivity. In addition, Kimura et al. (2013) investigated the SF selectivity of cat LGN neurons with bicuculline, a GABA receptor antagonist, reporting that neurons administered bicuculline iontophoretically exhibited more low-pass SF selectivity than those under the control condition. Thus, the antagonistic RF surround region of LGN neurons can be generated by both excitatory inputs from the RGCs and inhibitory inputs from local interneurons in the LGN and/or thalamic reticular nucleus.

Recently, Paik and Ringach (2011, 2012) suggested a model where the cortical orientation map takes origin from the retinal RF mosaic. More specifically, the retinal RF ON-OFF patterns, which are periodic but rotated and shifted with respect to one another, converge in the cortex, resulting in an orientation map of the cortex that has moiré interference patterns. Our current results suggest that there are convergent inputs in the retinogeniculate connections, thus we can assume the same orientation map in the LGN. In fact, Shou and Leventhal (1989) investigated the relationship between the preferred orientations and RF positions of cat LGN neurons, finding that near neurons prefer similar orientations. However, the orientation map in the cortex is unlikely to be directly inherited from the LGN because of geniculocortical convergent connections. Future studies are needed to clarify the relationships.

Another important finding was that a population of RGC-LGN pairs exhibited non-overlapped same-response-sign RFs (displaced same-response-sign projection, Figure 5B). It is possible that the non-overlapped same-response-sign and opposite-response-sign pairs are caused by pseudo projections from the RGCs. Rather than the simultaneously recorded RGC projecting to the LGN neuron, inter-retinally connected neighboring RGCs contributes to spike synchronization, which causes a non-overlapped or opposite-response-sign RF with the LGN neuron (Mastronarde, 1989) such that displaced inputs are the result of indirect connections. However, we found that connection strengths of neither input were lower than the near-placed same-sign inputs (Figures 6A,F). Thus, we conclude that the displaced projections are not indirect ones.

Another possibility is that the non-overlapped same-response-sign RFs were due to poor single unit isolation. Although we used strict criteria for single unit isolation as far as possible, the technical limitation did not allow us to completely eliminate the possibility of multi-unit recordings. If we recorded two RGCs with displaced RFs as a single unit, e.g. one RF overlapped with a target LGN RF and the other RF did not, the overlapped RF of RGC should also be reconstructed or extremely small efficacy/contribution values would be observed. Neither was observed in our results. In addition, the RFs of multi-unit activities will be larger than that of single-unit. However, we did not find such a trend (data not shown). Therefore, we concluded that it was unlikely that the non-overlapped same-response-sign RFs were due to poor single unit isolation.

Regardless, the functional significance of the displaced same-response-sign projection is not clear. Several previous studies have reported that the RF size of LGN neurons is not fixed but varies depending on the stimulus contrast both in cat (Nolt et al., 2004; Ozeki et al., 2004; Bonin et al., 2005; Sadakane et al., 2006) and primate (Kremers et al., 2001; Solomon et al., 2002). Therefore, if the efficacy and/or contribution of the displaced same-response-sign projection to the RF spatial structure of LGN neurons depend on the stimulus contrast, the displaced same-response-sign projection may contribute to the contrast dependent RF size of LGN neurons. This point will be addressed in our future studies.

### Efficacy and contribution, and RF property

We found that both efficacy and contribution were not significantly different between the same- and opposite-response-sign connections (Figures 6A,F). These results support our hypothesis that the opposite-response-sign inputs contribute to the generation of the antagonistic RF surround of the LGN neurons. Our data also suggest that there are some relationships between the connection strength and the connection types: SHORT (same-response-sign connections with near-placed RFs) had higher contributions with similar RFs (Figure 6J), LONG (same-response-sign connection with displaced RFs) became stronger when they shared less similar and more displaced RFs (Figures 6E,H), and opposite-response-sign connections with more displaced RFs became stronger (Figure 6C).

Previous studies have reported that retinogeniculate connections with closer or more similar RFs become stronger (Mastronarde, 1992; Usrey et al., 1999), which agrees with our results on SHORT. However, there are no or few studies that report the LONG and opposite-response-sign connections. The strengths of these connections were variable, in contradiction to our expectation. These connections probably have functions different to SHORT. As described above, the opposite-response-sign connections possibly enhance the antagonistic RF surround of the LGN neurons. Thus, the connections may be weighed in proportion to the inter-RF-centers distances; inputs near the RF center of the LGN neurons should be weak, and those far should be strong. Similarly, LONG may contribute to the contrast dependency of the size tuning exhibited in the LGN. To achieve this function, it may be efficient for LONG to have displaced and dissimilar RFs.

### Temporal RF structures of retinogeniculate-connected pairs

We found that projecting RGCs and their target LGN neurons exhibit similar temporal RF structures and that there seems to exist temporally-varied convergent inputs in retinogeniculate connections (Figures 9A,B). Hamamoto et al. (1994) investigated the TF selectivity of target LGN neurons and the corresponding projecting RGCs by recording S-potentials in cat LGN, finding that LGN neurons exhibited sharper band-pass TF selectivity than the projecting RGCs. Thus, these temporally-varied convergent inputs can facilitate temporal summation and induce a sharpening of the TF selectivity in connected neurons.

To summarize, LGN neurons have a spatiotemporal RF structure that have, compared to projecting RGCs similarly elongated, enhanced antagonistic surround and longer duration of response. In other words, LGN neurons can exhibit similar orientation selectivity, but sharper band-pass SF and TF selectivity compared to their projecting RGCs. Our results suggest that the orientation selectivity of LGN neurons is inherited from its primary projecting RGC, and the sharpened SF and TF selectivities are induced by convergent retinogeniculate connections. These connections can induce the staged visual image processing in the early visual system. Moreover, the resultant preferences may be inherited or enhanced in the visual cortex by geniculocortical convergent connections when the geniculate neurons projecting to a particular cortical neuron have the similar preferences. In a natural scene, there exist various sources of SF information such as low SF in the sky, middle SF in the contour of wood, and high SF in the textures of the road, and also various sources of TF information such as low TF in still life, middle TF in moving animals, and high TF in flickering light. Thus, retinogeniculate connections may conduct important visual image processing tasks that detect proper information while at the same time reducing noise.

## Author contributions

Hiromichi Sato designed and supervised the research, Naofumi Suematsu, Tomoyuki Naito, Tomomitsu Miyoshi, and Hajime Sawai collected the data, Naofumi Suematsu performed the analysis, and Naofumi Suematsu and Tomoyuki Naito wrote the paper.

### Conflict of interest statement

The authors declare that the research was conducted in the absence of any commercial or financial relationships that could be construed as a potential conflict of interest.
